# Recognition Strategies of Group 3 Innate Lymphoid Cells

**DOI:** 10.3389/fimmu.2014.00142

**Published:** 2014-04-01

**Authors:** Monica Killig, Timor Glatzer, Chiara Romagnani

**Affiliations:** ^1^Innate Immunity, Leibniz Institute, German Rheumatism Research Center, Berlin, Germany

**Keywords:** innate lymphoid cells, ILC3, RORγt, NKp44, NCR, AHR

## Abstract

During the early phase of an inflammatory response, innate cells can use different strategies to sense environmental danger. These include the direct interaction of specific activating receptors with pathogen-encoded/danger molecules or the engagement of cytokine receptors by pro-inflammatory mediators produced by antigen presenting cells in the course of the infection. These general recognition strategies, which have been extensively described for innate myeloid cells, are shared by innate lymphoid cells (ILC), such as Natural Killer (NK) cells. The family of ILC has recently expanded with the discovery of group 2 (ILC2) and group 3 ILC (ILC3), which play an important role in the defense against extracellular pathogens. Although ILC3 and NK cells share some phenotypic characteristics, the recognition strategies employed by the various ILC3 subsets have been only partially characterized. In this review, we will describe and comparatively discuss how ILC3 sense environmental cues and how the triggering of different receptors may regulate their functional behavior during an immune response.

## Introduction

Innate lymphoid cells (ILC) represent a family of innate effectors lacking recombination activating gene (RAG)-dependent rearranged antigen receptors and myeloid and dendritic cell (DC) markers. ILC are developmentally related as they all depend on the common cytokine receptor γ-chain as well as on the transcriptional repressor inhibitor of DNA binding 2 (ID2) for their development. ILC comprise of different effector populations characterized by distinct patterns of cytokine production and lineage-specific master transcription factors, tailored to their exclusive role in host defense. Thus, they closely resemble the heterogeneity of CD4^+^ T helper (T_H_) cell subsets. ILC can be grouped into three functionally distinct groups: group 1 ILC, among which Natural Killer (NK) cells are the main population, group 2 ILC (ILC2), and group 3 ILC (ILC3). ILC react promptly during infections or inflammatory responses and play an important role in tissue homeostasis, as well as in immune reactions against infectious microorganisms and transformed cells (1). While the recognition strategies employed by NK cells have been extensively investigated, the analysis of the receptors mediating the activation of ILC3 and ILC2 is still at the beginning. In this assay, we will review the current understanding of the signals able to activate and induce effector functions in ILC3 and discuss it in the context of NK cell recognition strategies.

## Group 3 ILC

Group 3 ILC or ILC3 are characterized by the expression of the transcription factor RORγt, which is critical for their development and function. A population of ILC3 emerges already during embryogenesis and corresponds to the previously described lymphoid-tissue inducer (LTi) cells, which are strictly required for the prenatal development of lymph nodes (LN) and Peyer’s patches (PP). In addition to RORγt, LTi cells express the IL-7 receptor (IL-7R or CD127), the stem cell factor (SCF) receptor c-kit (or CD117), IL-17, IL-22 and a number of tumor necrosis factor (TNF) family members, such as lymphotoxin (LT) α_1_β_2_ (2–8). While LT is crucial for the formation of secondary lymphoid structures, the role of IL-17 and IL-22 during embryogenesis is not clear, as both IL-22- and IL-17-deficient mice display normal lymphoid structures (9). ILC3 expressing IL-17 and IL-22 have also been identified after birth and are located mainly at mucosal surfaces (7, 10, 11). Mouse ILC3 can be dissected according to CD4 and CCR6 (11, 12). CCR6^+^ ILC3 share several features with LTi cells, being enriched in IL-17 and CD4 expression. Conversely, CCR6^−^ ILC3 do not express IL-17 or CD4 but produce IL-22. Among CCR6^−^ ILC3, a particular subset has been described, which is characterized by the expression of the NK cell activating receptor (actR) NKp46 and partial expression of NK1.1, producing not only IL-22, but also some IFN-γ (12–18). While all subsets depend for their development on RORγt, only CCR6^−^ ILC3 require the transcription factor T-bet, which is important for the differentiation toward NKp46^+^ ILC3 (12, 17, 18). Mouse ILC3 display a certain level of plasticity *in vitro* and *in vivo* (1, 19, 20). IL-7 and microflora have been reported to stabilize RORγt expression in small intestine (SI) lamina propria (LP) NKp46^+^ and NKp46^−^ ILC3, enabling them to maintain their phenotype of IL-22 producers. However, NKp46^+^ ILC3 from spleen or colon tend to lose RORγt expression while acquiring a full NK cell phenotype, which renders them almost undistinguishable from conventional NK cells. However, by using RORγt fate mapping (RORγt^fm^) to visualize NKp46^+^ cells derived from RORγt^+^ progenitors, it could be shown that a large fraction of colon LP and a minority of splenic NKp46^+^ cells actually represent ILC3, which have lost RORγt expression, as indicated by their phenotype of RORγt^fm+^ RORγt^−^ cells (20).

Similar to their mouse counterpart, human ILC3 are characterized by the expression of RORγt, IL-7R, c-kit, LTα_1_β_2_, and IL-22. Human fetal LTi, displaying LTi-like *in vitro* activity, express IL-17 and IL-22 but not CD4. After birth, human ILC3 can be mainly found not only in the gut LP but also in tonsils, from where they have been mostly isolated and characterized. Tonsil ILC3 are homogenously CD127^hi^, c-kit^+^, and LTα_1_β_2_^+^ and can be further dissected according to the expression of NKp44 and CD56 (7, 16, 21, 22). Although in one of the first reports, human IL-22-producing RORγt^+^ ILC3 were identified as Lin^−^ CD56^+^ NKp44^+^ cells and termed NK-22 (16), tonsil and gut LP CD56^−^ NKp44^+^ and CD56^+^ NKp44^−^ cells are also enriched in *RORC* transcripts. They display a partially overlapping phenotype compared to their CD56^+^ NKp44^+^ counterpart, thus suggesting that a large fraction of tonsil lineage (Lin)^−^ CD127^hi^ c-kit^+^ cells, which do not express the NK cell marker CD94 or the ILC2 marker CRTH2, might be bona fide ILC3 (21–23). It has been proposed that CD56^−^ ILC3 might represent the counterpart of CD4^+^ mouse LTi-like cells (7). However, some major differences between the mouse and human counterparts should be mentioned. In humans, ILC3 homogenously express CCR6, while lacking CD4 (7, 16). IL-17-producing ILC3 can be found among CD56^−^ NKp44^−^ ILC3 especially in fetal LN and gut LP of patients with Crohn’s disease, but not in tonsils (21, 24). Importantly, IL-22 expression is strictly confined to human NKp44^+^ ILC3 subsets (especially CD56^+^), which largely co-express NKp46 (16, 21, 22). Conversely, mouse IL-22 is preferentially expressed by NKp46^−^ ILC3 (25). Moreover, in contrast to the NKp46^+^ T-bet^+^ RORγt^+^ mouse ILC3 subset, T-bet and IFN-γ proteins are not produced *ex vivo* by tonsil-derived ILC3, although expression can be induced after *in vitro* culture (7, 16, 19, 22). In addition to the cytokines mentioned, human tonsil ILC3 have also been shown to express IL-26, GM-CSF, TNF, CCL20, LIF, IL-5, and IL-13. Intriguingly, human ILC3 produce consistent amounts of IL-2, whose *in vivo* function remains to be elucidated (7, 16, 19, 22, 26). Similarities and differences among human and mouse ILC3 subsets in comparison with splenic or blood NK cells are depicted in Table 1.

**Table 1:** **Phenotype of LTi, ILC3 subsets, and NK cells in human and mouse**.

	Mouse				Human			
	Fetal LTi	NCR^−^ILC3	NCR^+^ ILC3	Spleen NK cells	Fetal LTi	NCR^−^ILC3	NCR^+^ ILC3	Blood NK cells
**SURFACE MARKERS**								
CD127 (IL-7Rα)	+	+	+	−	+	+	+	lo/−
CD117 (c-Kit)	+	+	+	lo/−	+	+	+	lo/−
CD122 (IL-2Rβ)	ND	lo	lo	+	ND	lo	lo	+
NK1.1/CD161	−	−	lo/−	+	±	+	+	+/lo
CCR6	+	±	−	−	+	+	+	−
NKG2D	−	−	+	+	−	lo/−	lo/−	+
NKp30	NA	NA	NA	NA	±	±	+	+
NKp44	NA	NA	NA	NA	lo/−	−	+	+^a^
NKp46	−	−	+	+	−	±	+	+
CD56	NA	NA	NA	NA	lo/−	±	±	+
Perforin	−	−	−	+/lo	−	−	−	+/lo
CD16	ND	−	−	±	−	−	−	±
Ly49/KIR	−	−	−	±	−	−	−	±
CD94	ND	−	±	±	−	−	−	±
CD4	+	±	−	−	−	−	−	−
RANKL	+	+	+	lo/−	+	+	+	−
**CYTOKINES**								
TNF	+	+	+	+	+	+	+	+
IFN-γ	−	lo/−	lo/−	+	−	lo/−	lo/−	+
IL-17	+	±	−	−	+	lo/−	lo/−	−
IL-22	+	+	+	−	+	−	+	−
GM-CSF	ND	ND	ND	ND	ND	+	+	+
IL-2	ND	ND	ND	ND	ND	+	+	−
LTA/LTB	+	+	+	lo	+	+	+	lo
**TRANSCRIPTION FACTORS**								
T-bet	−	±	+	+	−	−	−	+
Eomes	−	−	−	+	−	−	−	+
RORγt	+	+	+	−	+	+	+	−
Gata-3	−	lo	lo	lo/−	lo/−	lo/−	lo/−	lo/−
AhR	+	+	+	−	+	+	+	−

*NCR^+^ refers to NKp46^+^ ILC3 in mouse and NKp44^+^ ILC3 in humans. + Indicates high level expression, − indicates no expression, ± indicates bimodal expression, and lo indicates low expression of the molecules according to published reports (1, 4, 6, 7, 11, 13, 14, 16–18, 21–23, 25, 27–35)*.

*NA, not applicable as gene is not conserved in the respective species. ND, not described*.

*^a^Expressed only on activated-NK cells*.

*AhR, aryl hydrocarbon receptor; CCR6, C–C chemokine receptor type 6; GM-CSF, granulocyte-macrophage-colony stimulating factor; IFN-γ, interferon-γ; IL, interleukin; KIR, killer-cell immunoglobulin-like receptor; LT, lymphotoxin; LTi cell, lymphoid-tissue inducer cell; NK cell, natural killer cell; RANKL, receptor activator of NF-κB ligand; RORγt, retinoic acid receptor-related orphan receptor-γt*.

## ILC3 Functions

### Role of ILC3 for the formation of secondary lymphoid organs and post-natal intestinal lymphoid clusters

LTi cells play a pivotal role during prenatal organogenesis of LN and PP. The most important effector molecules for lymphoid organogenesis are the TNF superfamily members, in particular LTα_1_β_2_ which, by triggering the LTβ-receptor (LTβR) on mesenchymal stem cells, induces the expression of adhesion molecules and chemokines, such as VCAM-1 and CXCL13. As a consequence, B cells, T cells, and DC can be recruited to form the LN (36). In addition, ILC3 are also required for post-natally developing intestinal lymphoid organs such as cryptopatches (CP) and isolated lymphoid follicles (ILF) (37). CP are lymphoid clusters located in the LP in between the gut crypts and comprise mainly of ILC3 surrounded by a wall of DC. When B cells are recruited to CP, ILF are formed, which represent important sites of T cell-independent IgA production. While the formation of CP is independent of the intestinal microbiota, formation of ILF requires signals from intestinal bacteria (27, 38–42).

### Maintenance of epithelial barrier function

ILC3 are critically involved in the maintenance of the barrier function, due to the production of cytokines instructing epithelial cell functions. Their ILC3 signature cytokine IL-22 is certainly a main mediator of the cross talk between epithelial cells, immune cells, and the commensal microflora. IL-22 belongs to the IL-10 family and binds the IL-22 receptor, a heterodimer comprising of IL-10R2 and IL-22R1, which is exclusively expressed on epithelial cells and signals mainly via STAT3. IL-22 induces the production of antimicrobial peptides and proteins, such as β-defensins, RegIIIβ and RegIIIγ, calgranulins S100A8, S100A9, and lipocalin-2 by epithelial cells (43–46). Furthermore, IL-22 induces epithelial cells to secrete elevated levels of mucus-associated molecules like Muc1, Muc3, Muc10, and Muc13, thus increasing the mucus production, whereby the translocation of commensal bacteria across the epithelial barrier during inflammation is reduced. *In vivo*, IL-22 induces the migration of epithelial cells and promotes wound healing during inflammation (47–50). Similar to its role in the intestine, IL-22 exerts tissue repairing functions also during liver inflammation (51–53). The ILC-mediated regulation of epithelial cell functions is strictly linked to their role in the containment of commensal and pathogenic microbes. Indeed, an intact ILC compartment is important for preventing peripheral dissemination and systemic inflammation of commensal bacteria such as *Alcaligenes species*, residing within PP and mesenteric LN of healthy humans and mice (54). Moreover, it was shown that IL-22 from ILC3 is important to contain the expansion of commensal-segmented filamentous bacteria (SFB), known to promote Th17 cells (55). On the other hand, microbiota can modulate production of IL-22 by ILC3 (25), although ILC3 development seems to be independent of gut flora or SFB (11, 56).

ILC3 also play an important role in the defense against pathogen infections, such as *Citrobacter rodentium*, a murine pathogen that models human enterohemorrhagic and enteropathogenic *Escherichia coli* infections. Protection against *C. rodentium* is mediated by IL-22, which is mainly produced by NKp46^−^ ILC3 in an IL-23-dependent manner (46, 57). Despite initial evidences for a role of NKp46^+^ ILC3 in the defense against *C. rodentium* infection (14, 16), these cells appear to be dispensable (12). Although T cells also importantly contribute to produce IL-22 after infection, early production of IL-22 is crucial for *C. rodentium* resistance, as *Il22*^-/-^ mice rapidly succumb within the first 8–12 days after infection (46, 58).

IFN-γ produced by T-bet-dependent CCR6^−^ ILC3 has been shown to contribute to the response against *Salmonella typhimurium* infection in mouse (12). Other reports have also recently outlined the importance of IL-17A and IL-17F production by ILC3 for the protection against mucosal *Candida* infections (59).

Due to their ability to modulate epithelial cell functions as well as to respond against commensal bacteria and pathogens, ILC3 also participate in the complex regulation of inflammatory bowel disease (IBD), displaying rather a dual role. Indeed, several investigators have suggested a protective role for IL-22, likely produced by ILC3, in innate and adaptive IBD models (47, 60). On the other hand, expression of IL-17 and IFN-γ from ILC3 has been implied to drive inflammation in innate IBD models, such as anti-CD40 or *Helicobacter hepaticus*-induced colitis (20, 61).

## Requirements for ILC3 Activation

Although many reports have clarified the functions of ILC3 and their role in the defense against pathogens, their receptor repertoire and the main signaling pathways able to trigger effector functions in ILC3 have not been extensively investigated. It was shown that, similar to NK cells, ILC3 can be mainly activated by cytokines released by the epithelium or antigen presenting cells (APC). More recently, receptors mediating a direct sensing of the environment by ILC3 have been also described. Here, we will revise the main findings concerning the recognition strategies that enable ILC3 to mediate their effector functions in response to perturbation of the epithelial barrier.

### Cytokine receptors

Both human and mouse ILC3 constitutively express the receptors for IL-7 and TSLP (IL-7R or CD127), IL-15/IL-2 (IL-2Rβγ), IL-23 (IL-23R), IL-1 (IL-1R), and SCF (c-kit). Among these cytokines, IL-7, TSLP, and SCF are required for ILC3 development and induce, together with IL-2/IL-15 and IL-1, proliferation of ILC3. Conversely, IL-23 and IL-1 play an important role in inducing ILC3 effector functions *in vitro* and *in vivo* (7, 8, 13, 16, 19, 62, 63). IL-23 is a heterodimer composed of subunits p19 and p40. The cellular sources are predominantly activated macrophages and DC (64). IL-23 was initially described as the main stimulus for induction of IL-22 expression in ILC3 (13, 16). Nevertheless, in IL-23p19-deficient mice or in wild-type mice treated with neutralizing anti-IL-23R antibody, the production of IL-22 by ILC3 is unaffected at steady state, suggesting that constitutive production of IL-22 by ILC3 is IL-23-independent (25). However, IL-23 becomes an important stimulus for innate production of IL-22 and IL-17 during *C. rodentium* and *H. hepaticus* infection (46, 61). In humans, IL-23 promotes IL-22 expression *in vitro*, together with IL-1 (65). Moreover, it induces IL-17 production in CD56^−^ ILC3 isolated from the gut LP of patients affected by Crohn’s disease (24). Altogether, these data imply the IL-23 axis as an important pathway of ILC3 activation during inflammation.

IL-1β is a key pro-inflammatory cytokine, which can be produced by different cell types (66). IL-1β does not only induce ILC3 proliferation, especially in combination with IL-7 and IL-2/IL-15 (19), but also induces the accumulation and activation of ILC3 during the course of *H. hepaticus* infection (67). IL-1β synergizes with IL-23 or IL-7 in stimulating ILC3 to produce IL-22 (22, 23). The importance of IL-1β for cytokine expression by ILC3 was also demonstrated by the decrease in basal as well as in IL-23-induced production of IL-22 observed in the presence of anti-IL-1R1-blocking antibodies or in mice deficient for IL-1R adaptor molecule, MyD88 (56).

In addition to their role in development of ILC3, IL-7, TSLP, and SCF also likely contribute to ILC3 maintenance post-natally (68). IL-7 enhances LTα_1_β_2_ expression (69, 70) and stabilizes *in vivo* the expression of RORγt on ILC3, thus preventing their conversion into IFN-γ-producing ILC3 (20). The main source of these cytokines is mainly non-hematopoietic cells such as fibroblasts, epithelial cells, and different types of stromal cells. Thus, it would be interesting to understand whether these cells get directly or indirectly instructed from ILC3 to produce IL-7, TSLP, and SCF.

### Environmental sensors

Apart from cytokine receptors, ILC3 are capable of directly recognizing environmental cues. Here, we will describe the main receptors that have been reported to enable ILC3 accomplishing this task.

#### Aryl hydrocarbon receptor

Both mouse and human ILC3 express the transcription factor aryl hydrocarbon receptor (AhR), belonging to the basic helix–loop–helix/Per–Arnt–Sim (bHLH/PAS) family of proteins. After engaging its ligand, AhR translocates from the cytoplasm to the nucleus where it pairs with AhR nuclear translocator (ARNT or HIF-1β) and then binds to xenobiotic response elements (XRE) present in the regulatory regions of AhR target genes (71, 72). Relevant target genes encode xenobiotic-metabolizing enzymes, including the cytochrome p450 superfamily members CYP1A1, CYP1A2, and CYP1B1. Several endogenous molecules, including metabolites of tryptophan and arachidonic acid have been shown to activate AhR. Among exogenous molecules functioning as AhR agonists, plant phytochemicals, such as polyphenols and glucosinolates as well as environmental toxins (dioxin) have been described. Moreover, bacterial metabolites have also been reported to display AhR agonist functions. Thus, AhR mainly acts as an environmental sensor (71, 73).

The number of post-natal ILC3 as well as the development of CP and ILF is drastically reduced in *Ahr*-deficient mice. Conversely, the number of fetal LTi and secondary organ development is not perturbed. The decrease in ILC3 observed in *Ahr*-deficient mice is not evident until the third week, suggesting that environmental stimuli may contribute to the differentiation, survival, and post-natal expansion of ILC3. Indeed, ILC3 from *Ahr*-deficient mice displayed lower expression of c-kit and IL-7R, as well as of the anti-apoptotic genes *Bcl2* and *Bcl2l1*. Moreover, IL-22 but not IL-17 expression by ILC3 in SI and colon was consistently reduced in *Ahr*-deficient mice (28–30). Despite the evidence of a central role of AhR in the maintenance and functions of ILC3, the endogenous and exogenous ligands driving this process still remain unclear. Kiss et al. have proposed a major role for AhR ligands derived from food components. Indeed, they could show that mice fed with phytochemical-free diets had a phenotype similar to *Ahr*-deficient mice (28). However, by using a different diet to feed mice, these data could not be confirmed by Lee et al. (29). Can ILC3 sense commensal bacteria via AhR? AhR ligands from bacterial metabolites can modulate ILC3 functions. It was recently shown that under conditions of unrestricted tryptophan availability, *Lactobacilli species* can produce an AhR ligand (indole-3-aldehyde) enhancing IL-22 expression in ILC3, which allows the survival of mixed microbial communities and provides colonization resistance to *Candida albicans* (74). In the future, it would be of great interest to understand whether ILC3 can employ AhR-dependent strategies to directly sense pathogens and produce effector cytokines.

#### Toll-like and other pattern recognition receptors

Pattern recognition receptors (PRR), such as Toll-like receptors (TLR), are mainly expressed on APC, such as macrophages or DC. Engagement of PRR by pathogen-derived ligands induces APC activation and production of pro-inflammatory cytokines (75). TLR2 and the C-type lectin receptor dectin-1 (or CLEC7A) bind β-glucans, i.e. structural cell wall polymers of fungi, and are implicated in the immune response to *Candida albicans* (76). Since both, the IL-17/IL-22 axis and ILC3 play a role in the defense against *Candida albicans* infection (59, 77), the role of PRR recognizing fungal components has been investigated. Injection of TLR2 and dectin-1 ligands can boost IL-17 and IL-22 production by mouse ILC3 as well as by γδ T cells (10, 78). However, while mouse IL-17-producing γδ T cells express both receptors, and directly respond to TLR2 stimulation, mouse ILC3 apparently lack TLR2 and are not directly activated by TLR2 ligands (26). By using quantitative RT-PCR, Crellin et al. observed broad expression of many TLR transcripts, including TLR1, 2, 5, 6, 7, and 9 by *ex vivo* isolated human ILC3 as well as by cloned ILC3 lines, although the degree of expression was still lower than on monocytes. Interestingly, only TLR2 agonists were able to induce cytokine production by human ILC3 in the presence of cytokines like IL-2, IL-15, and IL-23 (26). Thus, TLR2 engagement in ILC3 seems to act as a costimulus, rather than as a trigger on its own, as is the case when TLR agonists stimulate myeloid cells. This finding is in line with previous observations on other lymphocyte subsets, such as T cells and NK cells (79, 80). TLR2 triggering induces different responses depending on the cytokine employed as costimulation. TLR2 engagement induces IL-5 and IL-13 in the presence of exogenous IL-2 or IL-15, but not of IL-23. In contrast, IL-22 can be induced by either combination of signaling pathways (26). In light of its role in fungi recognition, TLR2 engagement might contribute to human ILC3 activation during *Candida* infection. Moreover, polymorphisms of TLR2 are linked with the disease phenotype in IBD. However, it remains to be established whether TLR2 activation of ILC3 plays a role *in vivo* (81).

#### NK cell activating receptors

Both human and mouse ILC3 subsets express NK cell actR, which have been described to mediate NK cell cytotoxicity and production of cytokines, such as IFN-γ and TNF, upon recognition of cognate cellular and viral ligands. NK cell actR include NK group 2, member D (NKG2D), DNAX accessory molecule (DNAM)-1, 2B4, CD94/NKG2C, and the natural cytotoxicity receptors (NCR), namely NKp46 (also known as NCR1 or CD335), NKp44 (also known as NCR2 or CD336), and NKp30 (also known as NCR3 or CD337). Human activating killer Ig-like receptors (KIR) and NKp80 or mouse Ly49 and NK1.1 also function as actR in NK cells (82, 83).

As previously mentioned, a subset of T-bet-dependent mouse ILC3 expresses NKp46 and has also been named NCR^+^ ILC3. NKp46^+^ ILC3 isolated from the SI LP of B6 mice largely co-express NKG2D and 2B4, while only a part of these cells co-express NK1.1 (15, 16). As engagement of actR induces effector functions in NK cells, actR triggering was also explored as a potential activating stimulus in ILC3. Engagement of NKp46, NK1.1, or 2B4 did not succeed in inducing IFN-γ or IL-22 expression in SI LP NKp46^+^ ILC3 (63). Conversely, NK1.1 but not NKp46 triggering was sufficient to drive IFN-γ and TNF, but not IL-22, expression in splenic NKp46^+^ RORγt^fm+^ RORγt^−^ ILC3 (22). A consistent fraction of human ILC3 derived from tonsils and gut LP expresses NKp44, NKp46, and NKp30, although to lower levels compared to NK cells. NKG2D or CD94/NKG2C is conversely not expressed by human ILC3. Thus, among NK cell actR, human ILC3 preferentially express NCR (7, 16, 21, 22, 65). In a recent study, we could show that among the different NK cell actR expressed by ILC3, namely NKp46, NKp30, and CD2, only engagement of NKp44 results in a strong cytokine response by ILC3 (22). Thus, the basic biology of NKp44 and its role on human ILC3 will be further discussed.

#### NKp44

NKp44 belongs to the NCR family, which represents type I membrane proteins of the immunoglobulin superfamily. NKp44 is not conserved between humans and mouse. In contrast to the other NCR, NKp44 is not expressed on resting human NK cells but it is up-regulated on their surface after IL-2 stimulation and upon engagement mediates the killing of susceptible tumor cell lines (84, 85). Conversely, NKp44 is detectable *ex vivo* on ILC3 and selectively marks the IL-22-producing subset in human tonsil and gut LP (16, 21, 22). NKp44 as well as NKp30 and NKp46 comprises of three domains: the extracellular ligand-binding domain, the transmembrane one, and a short cytosolic tail lacking intracellular signaling activity and therefore associating with ITAM-containing adaptor proteins (82, 83). Engagement of NCR results in recruitment and activation of zeta chain-associated protein kinase 70 (ZAP-70) and spleen tyrosine kinase (SYK), leading to the activation of several downstream signaling molecules, including phosphatidylinositol 3-kinase (PI3K) and phospholipase C (PLC)-γ1 or PLCγ2 (86). Unique among the NCR, NKp44 is coupled to a dimer of the ITAM-containing adaptor DNAX-activation protein (DAP)12 for downstream signal transduction and triggering of NKp44 in IL-2-activated NK cells leads to cytotoxicity of tumor target cells (84). Engagement of NKp44 in *ex vivo* isolated ILC3 is sufficient to induce cytokine production, demonstrating that ILC3 can directly sense the environment and be activated in the absence of pro-inflammatory cytokines (22). NKp44 triggering in *ex vivo* isolated ILC3 selectively induces the expression of TNF and IL-2 as well as a coordinated pro-inflammatory program, while cytokine stimulation (IL-23, IL-1, and IL-7) preferentially induces IL-22 and GM-CSF expression. Thus, ILC3 are able to switch between IL-22 or TNF production, depending on the triggering stimulus. However, combined engagement of NKp44 and cytokine receptors results in a strong synergistic effect both at transcriptome and protein level (22).

Which ligand is recognized by NKp44 expressed by NK cells or ILC3? Although several tumor cells and bacteria can bind NKp44–Ig fusion protein (87, 88), and NKp44 blocking via specific antibodies can decrease activated-NK cell-mediated cytolysis (84), as well as ILC3 cytokine production (22), the identity of NKp44 ligands triggering NK cells and ILC3 remains elusive. Discovering cellular ligands of the NCR still represents a great challenge, and only few cellular and viral ligands have been identified (89), such as the NKp30 ligands BAT3 (or BAG6) and B7-H6, which are expressed or released by tumor cells (90, 91). NKp44 has been described to bind to sialylated and sulfated cellular proteoglycans, hemagglutinin (HA) from influenza virus, and other viral HA-neuraminidase proteins, and HA engagement of NKp44 results in NK cell activation (92, 93). Surprisingly, influenza virus HA does not trigger NKp44-mediated cytokine expression in ILC3 (22). However, as sialic acid moieties attached to the stalk domain of NKp44 contribute directly to HA binding (93), glycosylation pattern of NKp44 expressed by NK cells or ILC3 should be analyzed. In addition, an inhibitory ligand of NKp44, namely proliferating cell nuclear antigen (PCNA), has also been reported (94). Very recently, a truncated form of the mixed lineage leukemia-5 (MLL5) protein has been described being an activating ligand of NKp44 and named NKp44L (95). Unlike MLL5, which is present in the nucleus and cytosol, NKp44L displays a unique C-terminal sequence that is required for its localization at the cell surface and its interaction with NKp44. NKp44L expression is present on the surface of several tumor cell lines, which are susceptible to NK cell-mediated lysis and their killing can be reduced using an anti-NKp44L antibody (95). As the nature of the NKp44 ligands eliciting inflammatory signatures in ILC3 remains elusive, it would be of great interest to test whether NKp44L could also trigger ILC3 cytokine production. In particular, it would be important to investigate whether, in addition to tumor cell lines, NKp44L would be also up-regulated on damaged or infected epithelial cells. NKp44 recognition of ligands expressed by microorganisms or intestinal epithelial cells could participate in fighting selected pathogens, restraining gut microflora, or even regulating epithelial cell homeostasis at steady state or during inflammation. In this context, it was recently reported that NKp44L is present on the surface of normal human articular chondrocytes (96). As ILC3 accumulate in the synovial fluid of patients affected by rheumatoid arthritis and can mediate proliferation of fibroblast-like synoviocytes in a TNF- and IL-22-dependent manner (97), the role of NKp44–NKp44L interactions in mediating this process should be investigated. Revealing the nature of NKp44 ligands relevant for ILC3 activation would enable us to better understand ILC3 functions in steady state and during inflammatory conditions.

## Conclusion

**Figure 1:**
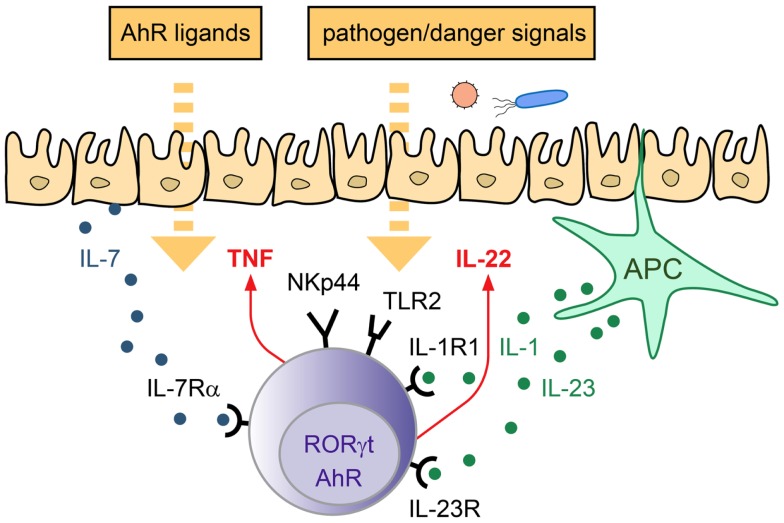
**Strategies of ILC3 to sense the environment**. LP-resident ILC3 can sense the environment via their receptors for cytokines, actR, AhR, and PRR. Depending on the triggering stimulus production of pro- and anti-inflammatory cytokines can be induced, which mediate the response to pathogens and promote tissue homeostasis/repair.

In conclusion, ILC3 are important innate effectors involved in the defense against extracellular pathogens as well as in the maintenance of the epithelial barrier. ILC3 can be activated directly after engagement of environmental sensors and/or in response to epithelial and APC-derived cytokines. Subsequently, they produce their signature cytokine IL-22 as well as other mediators, thus being able to modulate both, immune- as well as epithelial cell functions at mucosal interfaces (Figure 1).

## Conflict of Interest Statement

The authors declare that the research was conducted in the absence of any commercial or financial relationships that could be construed as a potential conflict of interest.
